# Supplementary material for: The temporal and genomic scale of selection following hybridization
Source: bioRxiv. 2023 May 26:2023.05.25.542345. Preprint. [Version 2] doi: 10.1101/2023.05.25.542345 (PMC10276902; doi:10.1101/2023.05.25.542345)
Supplement: Supplement 1 [file NIHPP2023.05.25.542345v2-supplement-1.pdf]

bioRxiv preprint doi: https://doi.org/10.1101/2023.05.25.542345; this version posted May 26, 2023. The copyright holder for this preprint (which was not certified by peer review) is the author/funder, who has granted bioRxiv a license to display the preprint in perpetuity. It is made available under aCC-BY 4.0 International license.

Supplementary Material

bioRxiv preprint doi: https://doi.org/10.1101/2023.05.25.542345; this version posted May 26, 2023. The copyright holder for this preprint (which was not certified by peer review) is the author/funder, who has granted bioRxiv a license to display the preprint in perpetuity. It is made available under aCC-BY 4.0 International license.

S1

As wavelets have not been widely used in population genetics, here we give some basic background on the Discrete Wavelet Transform. See Percival and Walden (2000) for a comprehensive treatment on theory and practical applications of the wavelet transform.

S1.1 Discrete Wavelet Transform (DWT)

The starting point for the DWT is a signal, i.e. a set of measurements that are ordered in space or time. In our case, these are measurements of ancestry state taken along a contiguous stretch of DNA, e.g. a single chromosome. We will write this ancestry signal as the vector $\mathbf{x}$ with entries $x(\ell)$,$\ell = 0, 1, 2, ..., L-1$, where L is the total length of the chromosome being analyzed. The values of ℓ must be evenly spaced in units defined by the resolution of measurement. For example, if ancestry state is measured at every nucleotide position, L would equal the number of nucleotide positions in the chromosome. If the ancestry state is instead measured in windows of 10kb along a chromosome, L would equal the number of such windows that fit on the chromosome. We will impose an additional restriction that L is a power of two (this will be relaxed later).

The DWT is an orthogonal transformation, where we project the ancestry signal onto a new set of coordinate axes. Instead of viewing the value of the ancestry signal at positions ℓ, we will instead view the data on a new set of coordinate axes where axes have the interpretation of measuring the magnitude of *change* in the ancestry signal at a particular genomic scale (defined in more detail below), and at a particular location. The DWT can be represented as a linear transformation as follows:

$$\mathbf{W} = \Psi\,\mathbf{x}. \tag{S1}$$

Above, $\mathbf{x}$ is a column vector, multiplied on the left by a square matrix Ψ to yield a new column vector $\mathbf{W}$, whose entries are the wavelet coefficients. The rows of Ψ are thus the basis vectors of the new coordinate system. The first $L-1$ of these are known as *wavelets*, and the Lth is known as the scaling filter. These are defined below. In practice, the DWT is accomplished using a fast algorithm known as the pyramid algorithm (Percival and Walden 2000).

S1.1.1 Haar wavelets

Each wavelet $\psi(\ell)$ is written as a function of the genomic position ℓ, and so is defined on the same set of values as $x(\ell)$. When the values of $\psi(\ell)$ are plotted against the values of ℓ, the function resembles a wave in the sense that the values grow and decay. However the wave is ‘small’ in the sense that this oscillatory behaviour may be limited to a subset of the domain. For example, a sine wave oscillates infinitely over its domain and so does not obey this second property. This notion is formalized in the following two properties:

$$\sum_{\ell=0}^{L-1} \psi(\ell) = 0, \tag{S2a}$$

$$\sum_{\ell=0}^{L-1} \psi(\ell)^2 = 1. \tag{S2b}$$

Any function with these properties could be used as a wavelet. However, for a given DWT analysis, all of the wavelets in the matrix Ψ belong the same family. The wavelets of the same family are all related through a set of shifting and scaling operations. From here on we specifically focus on Haar wavelets. A Haar wavelet is a piecewise constant function that is non-zero over a finite portion of the sequence, and has a single transition from positive to negative values (see Fig. 1 for examples). Each Haar wavelet is indexed by two parameters, j and k. (Note in the main text we index wavelets using λ and i in place of j and k for ease of description, but the two notations are equivalent). The parameter j, or the level of the wavelet, corresponds with the length of sequence over which the Haar wavelet is non-zero, and the parameter k, or shift, corresponds with where in the sequence the non-zero portion is located. Formally, the set of Haar wavelets used in the DWT is defined as follows:

bioRxiv preprint doi: https://doi.org/10.1101/2023.05.25.542345; this version posted May 26, 2023. The copyright holder for this preprint (which was not certified by peer review) is the author/funder, who has granted bioRxiv a license to display the preprint in perpetuity. It is made available under aCC-BY 4.0 International license.

$$\psi_{j,k}(\ell) = \begin{cases} 0 & \ell < (k-1)2^j \text{ or } \ell \geq k2^j \\ 2^{-j/2} & (k-1)2^j \leq \ell < (k-1)2^j + 2^{j-1} \\ -2^{-j/2} & (k-1)2^j + 2^{j-1} \leq \ell < k2^j \end{cases} \tag{S3}$$

where $j = 1, 2, ... \log_2 L$ and $k = 1, ..., L/2^j$. Haar wavelets of level j only take non-zero values in one interval of the domain, which is of length 2^λ. We define the *scale* of a wavelet, λ to be equal to half of this interval, i.e. $\lambda = 2^{j-1}$, so we can say the wavelet changes from positive to negative over a scale of λ (this definition corresponds with the description of λ in the main text). Thus, the set of scales that the DWT yields information about are a dyadic series, that is, successive scales differ by a factor of two. We also see that the number of wavelets at a given level, $L/(\lambda + 1)$, is simply the number of wavelets at that level that fit onto the chromosome with no overlap in where they take non-zero values. Because of our restriction that L must be a power of two, this is always whole number. Thus, the number of wavelets decreases by two for successively larger scales. If we order the wavelets at a given level with their non-zero portions arranged adjacent from left to right, $\psi_{j,k}$ is the kth wavelet in this ordered set. In this sense, the parameter k describes where in the domain the non-zero portion of the wavelet is located.

The definition given above satisfies a third property of the wavelets used in the DWT, which is that they form an orthogonal set. Formally,

$$\sum_{\ell=0}^{L-1} \psi_{j,k}(\ell)\psi_{j',k'}(\ell) = 0 \quad \text{if } j \neq j' \text{ or } k \neq k'. \tag{S4}$$

As a toy example, for a very small chromosome with $L = 8$, the wavelets used in the DWT are:

$$\begin{aligned} \psi_{1,1} &= \left\{\frac{-1}{\sqrt{2}}, \frac{1}{\sqrt{2}}, 0, 0, 0, 0, 0, 0\right\}, \quad \psi_{1,2} = \left\{0, 0, \frac{-1}{\sqrt{2}}, \frac{1}{\sqrt{2}}, 0, 0, 0, 0\right\}, \\ \psi_{1,3} &= \left\{0, 0, 0, 0, \frac{-1}{\sqrt{2}}, \frac{1}{\sqrt{2}}, 0, 0\right\}, \quad \psi_{1,4} = \left\{0, 0, 0, 0, 0, 0, \frac{-1}{\sqrt{2}}, \frac{1}{\sqrt{2}}\right\}, \\ \psi_{2,1} &= \left\{\frac{-1}{2}, \frac{-1}{2}, \frac{1}{2}, \frac{1}{2}, 0, 0, 0, 0\right\}, \quad \psi_{2,2} = \left\{0, 0, 0, 0, \frac{-1}{2}, \frac{-1}{2}, \frac{1}{2}, \frac{1}{2}\right\}, \\ \psi_{3,1} &= \left\{\frac{-1}{\sqrt{8}}, \frac{-1}{\sqrt{8}}, \frac{-1}{\sqrt{8}}, \frac{-1}{\sqrt{8}}, \frac{1}{\sqrt{8}}, \frac{1}{\sqrt{8}}, \frac{1}{\sqrt{8}}, \frac{1}{\sqrt{8}}\right\}. \end{aligned} \tag{S5}$$

There is additionally one *scaling filter*, $G(\ell)$, which is constant valued, $G(\ell) = 1/\sqrt{L}$ for $l = 0, 1, ...L-1$. Because of this, G does not obey property S2a, but it does obey properties S2 and S4.

S1.1.2 Wavelet coefficients

We have now seen that the rows of Ψ are the wavelets $\psi_{\lambda,k}(\ell)$, along with the scaling filter $G(\ell)$ as described above. The nth entry of $\mathbf{W}$ is an inner product between the nth row of Ψ and the vector $\mathbf{x}$. The inner product between a wavelet and $\mathbf{x}$ yields a wavelet coefficient. Because the mean of any wavelet is zero, a particular wavelet coefficient is proportional to the covariance between the ancestry signal and the associated wavelet:

$$w_{j,k} = \sum_{\ell=0}^{L-1} x(\ell)\psi_{j,k}(\ell) = L\text{cov}(x(\ell), \psi_{j,k}(\ell)). \tag{S6}$$

The inner product of the scaling filter and $\mathbf{x}$ produces the *scaling coefficient* which is proportional to the mean of $x(\ell)$:

$$v = \sum_{\ell=0}^{L-1} x(\ell)G(\ell) = \bar{\mathbf{x}}\sqrt{L}. \tag{S7}$$

bioRxiv preprint doi: https://doi.org/10.1101/2023.05.25.542345; this version posted May 26, 2023. The copyright holder for this preprint (which was not certified by peer review) is the author/funder, who has granted bioRxiv a license to display the preprint in perpetuity. It is made available under aCC-BY 4.0 International license.

If we sort the rows Ψ by increasing j and then k, and finally the scaling filter G, we can expand the matrix multiplication of Eqn. S1 using Haar wavelets:

$$\mathbf{W} = \begin{bmatrix} w_0 \\ \vdots \\ w_{\frac{L}{2}} \\ \vdots \\ w_{\frac{L}{2}+\frac{L}{4}} \\ \vdots \\ w_{\frac{L}{2}+\frac{L}{4}+\frac{L}{8}} \\ \vdots \\ w_{L-2} \\ w_{L-1} \end{bmatrix} = \begin{bmatrix} \frac{1}{\sqrt{2}}\big(x(1) - x(0)\big) \\ \vdots \\ \frac{1}{2}\big(\sum_{\ell=2}^{3} x(\ell) - \sum_{\ell=0}^{1} x(\ell)\big) \\ \vdots \\ \frac{1}{\sqrt{8}}\big(\sum_{\ell=4}^{7} x(\ell) - \sum_{\ell=0}^{3} x(\ell)\big) \\ \vdots \\ \frac{1}{8}\big(\sum_{\ell=8}^{15} x(\ell) - \sum_{\ell=0}^{7} x(\ell)\big) \\ \vdots \\ \frac{1}{\sqrt{L}}\big(\sum_{\ell=\frac{L}{2}}^{L-1} x(\ell) - \sum_{\ell=0}^{\frac{L}{2}-1} x(\ell)\big) \\ \frac{1}{\sqrt{L}}\sum_{\ell=0}^{L-1} x(\ell) \end{bmatrix}. \tag{S8}$$

From this we can see that the magnitude of a particular wavelet coefficient produced with a Haar wavelet is proportional to the magnitude of change occurring between two adjacent windowed averages of the ancestry signal, where the window length is the scale of the associated wavelet.

Thus, we are now viewing $\mathbf{x}$ in a new set of coordinates that describe (i) fluctuations around that mean value of $\mathbf{x}$ and different scales and locations, and (ii) the mean value of $\mathbf{x}$ itself.

S1.1.3 DWT wavelet variance decomposition

We can use the wavelet coefficients to understand what portion of the total variance across loci in ancestry state can be attributed to variation at different genomic scales. Since the DWT is an orthonormal transformation, we have the following property: $\sum_\ell x(\ell)^2 = \sum_\ell W(\ell)^2$. Using this property and the property that $v = \overline{\mathbf{x}}\sqrt{L}$, we can write the sample variance of $\mathbf{x}$ as a sum of contributions from different scales, indexed by λ:

$$\begin{aligned} \hat{\sigma}^2_{\mathbf{x}} &= \frac{1}{L}\sum_\ell x(\ell)^2 - \overline{\mathbf{x}}^2 = \frac{1}{L}\sum_\ell \mathbf{W}(\ell)^2 - \overline{\mathbf{x}}^2 \\ &= \frac{1}{L}\left(\sum_\lambda\sum_k w^2_{\lambda,k} + v^2\right) - \overline{\mathbf{x}}^2 \\ &= \frac{1}{L}\sum_\lambda\sum_k w^2_{\lambda,k} \\ &= \sum_\lambda \hat{\sigma}^2_\lambda. \end{aligned} \tag{S9}$$

S1.1.4 Wavelet covariance decomposition

Analogous to the wavelet variance, we can compute the wavelet covariance at scale λ for two signals x and y:

$$\widehat{\mathrm{cov}}_\lambda(x,y) = \frac{1}{L}\sum_k w_{x,\lambda,k} w_{y,\lambda,k} \tag{S10}$$

and for the DWT the sum of these gives an exact decomposition of the total covariance:

$$\widehat{\mathrm{cov}}(x,y) = \sum_\lambda \widehat{\mathrm{cov}}_\lambda(x,y) \tag{S11}$$

bioRxiv preprint doi: https://doi.org/10.1101/2023.05.25.542345; this version posted May 26, 2023. The copyright holder for this preprint (which was not certified by peer review) is the author/funder, who has granted bioRxiv a license to display the preprint in perpetuity. It is made available under aCC-BY 4.0 International license.

S1.2 Maximal Overlap Discrete Wavelet Transform (MODWT)

The DWT is restricted to signals that are powers of two, and the coefficients produced by the DWT are not shift-invariant. In other words, the set of coefficients resulting from performing the DWT on a shifted signal (moving every value by the same amount in one direction, and treating the signal as circular) are not the same set of coefficients resulting from the DWT on the original signal.

The MODWT is a related transform that is both well-defined for signals of any length and shift invariant (Percival and Walden 2000). In essence, it results from circularly shifting $\mathbf{x}$ and performing the DWT, resulting in a vector of wavelet coefficients $\mathbf{W}_\lambda$ of length L for each λ, representing localized changes in adjacent averages taken in windows of size λ, as well as one vector of scaling coefficients $\mathbf{V}$ of length L corresponding to average values of the signal in windows of size $\lfloor \log_2 L \rfloor$. One consequence of this procedure is that the first λ elements of $\mathbf{W}_\lambda$ and $\mathbf{V}$ are so-called *boundary coefficients* that describe changes between discontiguous portions at the beginning and end of the signal. Different approaches for dealing with these coefficients when calculating wavelet variances are available (described in Percival and Walden). In our application, we have omitted boundary coefficients in our calculation of wavelet variances, but including boundary coefficients produced qualitatively similar results.

Also in contrast to the DWT, the MODWT is not an orthogonal transformation, as there is redundancy among neighboring wavelet coefficients within each level. In other words, wavelet coefficients will often be auto-correlated along the signal. This does not however bias the estimates of the wavelet variances, and we use MODWT as it produces less noisy estimates of wavelet variances. Similar to the DWT, the MODWT gives a complete decomposition of the sample variance as follows:

$$\hat{\sigma}^2_{\mathbf{x}} = \frac{1}{L}\sum_{\ell} x(\ell)^2 - \overline{\mathbf{x}}^2 = \frac{1}{L}\sum_{\lambda}\sum_{\ell} W_\lambda(\ell)^2 + \frac{1}{L}\sum_{\ell} V(\ell)^2 - \overline{\mathbf{x}}^2. \tag{S12}$$

We define the portion $\hat{\sigma}^2_{\mathbf{V}} = \frac{1}{L}\sum_\ell V(\ell)^2 - \overline{\mathbf{x}}^2$ as the scaling variance, and note that unlike the wavelet variances, it's calculation requires subtracting the squared mean of coefficients. The scaling variance has a different interpretation than the wavelet variances as it is associated with variance of average values of the signal taken in windows of size $\lfloor \log_2 L \rfloor$, rather than being a variance associated with changes. Similarly, in computing the covariance decomposition shown above for the DWT, the exact decomposition includes the covariance of scaling coefficients for x and y. In contrast to the wavelet covariances, the scaling covariance subtracts the product of the means of the scaling coefficients of the two signals.

bioRxiv preprint doi: https://doi.org/10.1101/2023.05.25.542345; this version posted May 26, 2023. The copyright holder for this preprint (which was not certified by peer review) is the author/funder, who has granted bioRxiv a license to display the preprint in perpetuity. It is made available under aCC-BY 4.0 International license.

S2

In this section we derive expectations of the wavelet variance for ancestry state along the genome under a neutral single-pulse model of hybridization.

S2.1 Expectation for wavelet variance of sample mean ancestry

We write the mean ancestry at position ℓ in a sample of size M chromosomes as follows

$$\overline{x}(\ell) = \frac{1}{M}\sum_{i=1}^{M} \mathbb{1}_i(\ell). \tag{S13}$$

where $\mathbb{1}_i(\ell)$ is an indicator random variable; $\mathbb{1}_i(\ell) = 1$ if haplotype i carries an introgressed allele at locus ℓ and is zero otherwise. We will later refer to the 'recipient' and 'donor' populations as populations 0 and 1, respectively, but the choice is arbitrary with respect to the admixture proportions.

From Eqn. S9, the expected contribution to the total variance of mean ancestry $\overline{x}(\ell)$ along the sequence associated with level λ is given by

$$\mathbb{E}[\hat{\sigma}^2_\lambda(\overline{x})] = \mathbb{E}\Big[\frac{1}{L}\sum_k w^2_{\lambda,k}(\overline{x})\Big] = \frac{1}{L}\sum_k \mathbb{E}\Big[w^2_{\lambda,k}(\overline{x})\Big] = \frac{1}{L}\sum_k \mathbb{E}\left(\sum_\ell \psi_{\lambda,k}(\ell)\overline{x}(\ell)\right)^2. \tag{S14}$$

We can approximate the sum over the L loci as a continuous integral over a region of length L. Then,

$$\begin{aligned}
\mathbb{E}[w^2_{\lambda,k}(\overline{x})] &= \mathbb{E}\left[\left(\int_{\ell=0}^{L} \psi_{\lambda,k}(\ell)\,\overline{x}(\ell)\,d\ell\right)^2\right] \\
&= \mathbb{E}\left[\int_{\ell=0}^{L} \psi_{\lambda,k}(\ell)\,\overline{x}(\ell)\,d\ell \int_{\ell'=0}^{L} \psi_{\lambda,k}(\ell')\,\overline{x}(\ell')\,d\ell'\right] \\
&= \int_{\ell=0}^{L}\int_{\ell'=0}^{L} \psi_{\lambda,k}(\ell)\,\psi_{\lambda,k}(\ell')\,\mathbb{E}\big[\overline{x}(\ell)\overline{x}(\ell')\big]\,d\ell' d\ell \\
&= \int_{\ell=0}^{L}\int_{\ell'=0}^{L} \psi_{\lambda,k}(\ell)\,\psi_{\lambda,k}(\ell')\,\mathbb{E}\left[\left(\frac{1}{M}\sum_{i=1}^{M}\mathbb{1}_i(\ell)\right)\left(\frac{1}{M}\sum_{i=1}^{M}\mathbb{1}_i(\ell')\right)\right] d\ell' d\ell \\
&= \int_{\ell=0}^{L}\int_{\ell'=0}^{L} \psi_{\lambda,k}(\ell)\,\psi_{\lambda,k}(\ell')\frac{1}{M^2}\left(\sum_{i=1}^{M}\mathbb{E}\big[\mathbb{1}_i(\ell)\,\mathbb{1}_{i,\ell'}\big] + \sum_{i=1}^{M}\sum_{j\neq i}^{M}\mathbb{E}\big[\mathbb{1}_i(\ell)\,\mathbb{1}_j(\ell')\big]\right) d\ell' d\ell \\
&= \int_{\ell=0}^{L}\int_{\ell'=0}^{L} \psi_{\lambda,k}(\ell)\,\psi_{\lambda,k}(\ell')\left(\frac{1}{M}\mathbb{E}\big[\mathbb{1}_i(\ell)\,\mathbb{1}_i(\ell')\big] + \frac{M-1}{M}\mathbb{E}\big[\mathbb{1}_i(\ell)\,\mathbb{1}_j(\ell')\big]\right) d\ell' d\ell \\
&= \frac{1}{M}\int_{\ell=0}^{L}\int_{\ell'=0}^{L} \psi_{\lambda,k}(\ell)\,\psi_{\lambda,k}(\ell')\mathbb{E}\big[\mathbb{1}_i(\ell)\,\mathbb{1}_i(\ell')\big] d\ell' d\ell \, + \\
&\quad \frac{M-1}{M}\int_{\ell=0}^{L}\int_{\ell'=0}^{L} \psi_{\lambda,k}(\ell)\,\psi_{\lambda,k}(\ell')\mathbb{E}\big[\mathbb{1}_i(\ell)\,\mathbb{1}_j(\ell')\big] d\ell' d\ell
\end{aligned} \tag{S15}$$

We can interpret $\mathbb{E}[\mathbb{1}_i(\ell)\,\mathbb{1}_j(\ell')]$ as the probability that haplotype i inherits from the donor population at locus ℓ and individual j inherits from the donor population at locus ℓ'. From (S15), we also see that for a general statistic, the wavelet variance of the sample mean is equal to a weighted average: $1/M$ times the average wavelet variance for single chromosomes, plus $(M-1)/M$ times the average wavelet covariance of pairs of chromosomes, where M is the sample size.

S2.2 Neutral case.

Under neutrality, both of the expectations in (S15) will only depend on the distance between ℓ and ℓ', and not their exact locations, so there is no dependence on k. Thus, we have

bioRxiv preprint doi: https://doi.org/10.1101/2023.05.25.542345; this version posted May 26, 2023. The copyright holder for this preprint (which was not certified by peer review) is the author/funder, who has granted bioRxiv a license to display the preprint in perpetuity. It is made available under aCC-BY 4.0 International license.

$$\mathbb{E}[\hat{\sigma}^2_\lambda(\overline{x})] = \frac{1}{L}\sum_k \mathbb{E}\Big[w^2_{\lambda,k}(\overline{x})\Big] = \frac{1}{2^\lambda}\mathbb{E}\Big[w^2_\lambda(\overline{x})\Big]. \tag{S16}$$

The two expectations in (S15) can then be calculated under a coalescent model using a two state continuous time Markov chain that runs backward in time from the present. Let state $X = 1$ represent the alleles at loci ℓ and ℓ' being present on the same haplotype, and state $X = 2$ represents the two alleles being present on separate haplotypes. The transition rate matrix is given by the coalescent approximation with time scaled to units of $2N$ generations ($\tau = 2Nt$),

$$Q = \begin{bmatrix} -2Nr|\ell'-\ell| & 2Nr|\ell'-\ell| \\ 1 & -1 \end{bmatrix}.$$

The transition probability matrix is given by

$$\begin{bmatrix} \mathbb{P}\big(X(\tau)=1 \mid X(0)=1\big) & \mathbb{P}\big(X(\tau)=2 \mid X(0)=1\big) \\ \mathbb{P}\big(X(\tau)=1 \mid X(0)=2\big) & \mathbb{P}\big(X(\tau)=2 \mid X(0)=2\big) \end{bmatrix} = e^{Q\tau} = Ae^{D\tau}A^{-1}$$

with D containing the eigenvalues of Q on the diagonal and the columns of A containing the corresponding right eigenvectors so that

$$Ae^{D\tau}A^{-1} = \begin{bmatrix} 1 & 1 \\ \frac{-1}{2Nr|\ell'-\ell|} & 1 \end{bmatrix} \begin{bmatrix} e^{-\tau(1+2Nr|\ell'-\ell|)} & 0 \\ 0 & 1 \end{bmatrix} \begin{bmatrix} \frac{-1}{1+2Nr|\ell'-\ell|} & \frac{-2Nr|\ell'-\ell|}{1+2Nr|\ell'-\ell|} \\ \frac{1}{1+2Nr|\ell'-\ell|} & \frac{2Nr|\ell'-\ell|}{1+2Nr|\ell'-\ell|} \end{bmatrix}.$$

For an initial admixture proportion of α, two alleles present on the same haplotype at the time of admixture come from the donor population with probability α, while two alleles present on different haplotypes at the time of admixture come from the donor population with probability α^2. We then have

$$\begin{aligned} \begin{bmatrix} \mathbb{E}[\mathbb{1}_i(\ell)\,\mathbb{1}_i(\ell')] \\ \mathbb{E}[\mathbb{1}_i(\ell)\,\mathbb{1}_j(\ell')] \end{bmatrix} &= Ae^{D\tau}A^{-1}\begin{bmatrix} \alpha \\ \alpha^2 \end{bmatrix} \\ &= \begin{bmatrix} \alpha\Big(\frac{1+2Nr|\ell'-\ell|e^{-\tau(1+2Nr|\ell'-\ell|)}}{1+2Nr|\ell'-\ell|}\Big) + \alpha^2\Big(\frac{2Nr|\ell'-\ell|(1-e^{-\tau(1+2Nr|\ell'-\ell|)})}{1+2Nr|\ell'-\ell|}\Big) \\ \alpha\Big(\frac{1-e^{-\tau(1+2Nr|\ell'-\ell|)}}{1+2Nr|\ell'-\ell|}\Big) + \alpha^2\Big(\frac{2Nr|\ell'-\ell|+e^{-\tau(1+2Nr|\ell'-\ell|)}}{1+2Nr|\ell'-\ell|}\Big) \end{bmatrix}. \end{aligned} \tag{S17}$$

Note in the above that if we set $t = 0$ we get $\mathbb{E}[\mathbb{1}_i(\ell)\,\mathbb{1}_i(\ell')] = \alpha$ and $\mathbb{E}[\mathbb{1}_i(\ell)\,\mathbb{1}_j(\ell')] = \alpha^2$ as expected. In this case, since $\psi_{\lambda,k}(\ell)$ integrates to zero, the integrand in (S15) becomes an even function with no dependence on the distance between loci and we get $\mathbb{E}[\hat{\sigma_\lambda}^2] = 0$. This makes intuitive sense; with no time for recombination after the initial admixture, there is zero variance at all spatial scales as introgressed alleles are in complete linkage. In the limit as $N \to \infty$, both expectations depend only on the recombination process and we have

$$\lim_{N\to\infty}\begin{bmatrix} \mathbb{E}[\mathbb{1}_i(\ell)\,\mathbb{1}_i(\ell')] \\ \mathbb{E}[\mathbb{1}_i(\ell)\,\mathbb{1}_j(\ell')] \end{bmatrix} = \begin{bmatrix} \alpha e^{-rt|\ell'-\ell|} + \alpha^2(1-e^{-rt|\ell'-\ell|}) \\ \alpha^2 \end{bmatrix}. \tag{S18}$$

In this particular case the wavelet variance of the mean is equivalent to the wavelet variance for a single haplotype, since drift cannot induce any covariance among haplotypes.

These expectations in general can also be found under an any demographic model where the population change size changes through time as a piecewise constant function of time. The length of the total time interval between the present and the time of admixture, τ, is subdivided into n component internals of lengths $\tau_1, \tau_2, ...\tau_n$ which sum to τ. The probability transition matrix over the entire interval is the result of multiplying probability transition matrices for subsequent subintervals on the right (moving backward in time):

$$\begin{bmatrix} \mathbb{P}\big(X(\tau)=1 \mid X(0)=1\big) & \mathbb{P}\big(X(\tau)=2 \mid X(0)=1\big) \\ \mathbb{P}\big(X(\tau)=1 \mid X(0)=2\big) & \mathbb{P}\big(X(\tau)=2 \mid X(0)=2\big) \end{bmatrix} = e^{Q\tau_1}e^{Q\tau_2}e^{Q\tau_3}\cdots e^{Q\tau_n}.$$

bioRxiv preprint doi: https://doi.org/10.1101/2023.05.25.542345; this version posted May 26, 2023. The copyright holder for this preprint (which was not certified by peer review) is the author/funder, who has granted bioRxiv a license to display the preprint in perpetuity. It is made available under aCC-BY 4.0 International license.

S2.3 Discrepancy for early generations

In early generations after admixture, the coalescent provides a poor model for the distribution of ancestry tract lengths, because the fixed pedigree constrains which ancestors can be inherited from (Liang and Nielsen 2014). We thus expect error in the calculations above for the first few generations after hybridization. To illustrate this, we calculate the expected wavelet variance for the F2 generation, assuming no selection or assortative mating in the F1 generation. To match our Wright-Fisher simulation framework, we imagine that two populations mix with proportions a and $1-\alpha$. In the F1 generation, individuals are either descended from a mating between two individuals from the same source population (events A, B with probabilities α^2 and $(1-\alpha)^2$, respectively), or they are F1s (event C) with probability $2\alpha(1-\alpha)$. We then have

$$\mathbb{E}[\mathbb{1}_i(\ell)\mathbb{1}_i(\ell)] = \mathbb{E}[\mathbb{1}_i(\ell)\mathbb{1}_i(\ell)|A]\mathbb{P}(A) + \mathbb{E}[\mathbb{1}_i(\ell)\mathbb{1}_i(\ell)|B]\mathbb{P}(B) + \mathbb{E}[\mathbb{1}_i(\ell)\mathbb{1}_i(\ell)|C]\mathbb{P}(C) \tag{S19}$$

Using Haldane's map function for the probability that a chromosome produced through meiosis in an F1 is not recombinant at ℓ and ℓ', this becomes

$$\mathbb{E}[\mathbb{1}_i(\ell)\mathbb{1}_i(\ell)] = \alpha^2 + \frac{1}{2}2\alpha(1-\alpha)\big(1 - \frac{1}{2}(1 - e^{-2|\ell-\ell'|})\big) \tag{S20}$$

For $\mathbb{E}[\mathbb{1}_i(\ell)\mathbb{1}_j(\ell)]$, we only need to consider whether the lineages coalesce in the previous generation:

$$\mathbb{E}[\mathbb{1}_i(\ell)\mathbb{1}_j(\ell)] = \frac{1}{2N}\alpha + \frac{2N-1}{2N}\alpha^2. \tag{S21}$$

Using the expectations in Eqns. S20 and S21 in the integrand for the wavelet variance calculation, we find that this provides a much better fit for the simulated wavelet variance in the F2 generation compared to the coalescent model specified by Eqn. S17 (Fig. S1).

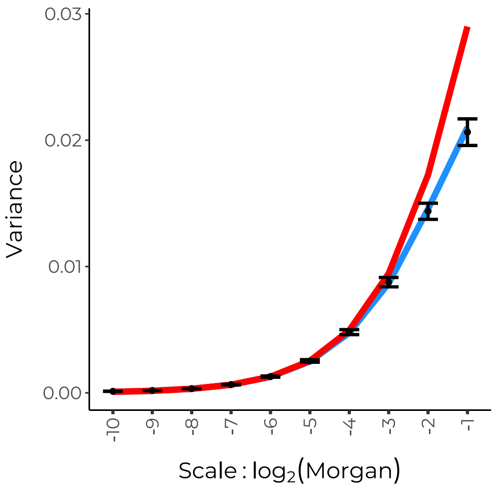

Figure S1: Black points and error bars show mean wavelet variances and 95% confidence intervals for ancestry state along single chromosomes in the F2 generation from 100 replicate simulations of a 50/50 mixture between two populations. Red line shows expected wavelet variance using the coalescent model. Blue line shows expected wavelet variance calculated for the F2 generation by conditioning on the pedigree.

bioRxiv preprint doi: https://doi.org/10.1101/2023.05.25.542345; this version posted May 26, 2023. The copyright holder for this preprint (which was not certified by peer review) is the author/funder, who has granted bioRxiv a license to display the preprint in perpetuity. It is made available under aCC-BY 4.0 International license.

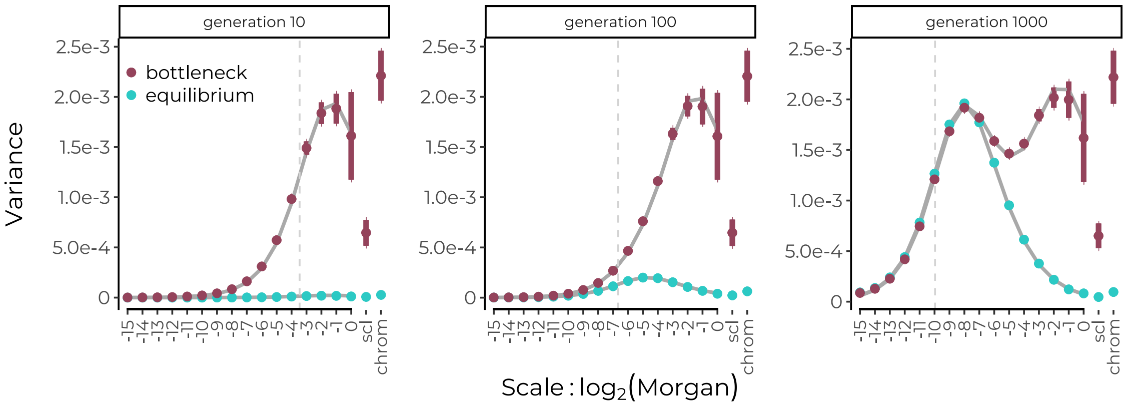

Figure S2: Simulated power spectrum of ancestry proportion in a hybrid population undergoing genetic drift compared to theoretical expectations. We simulated (using SliM, Haller and Messer, 2019) a population of constant size 2N=20000 (light blue) and a population that undergoes a bottleneck to 2N=200 for just the first 10 generations of recombination in hybrids, then expands to 2N=20000 (maroon). Points and error bars show means and 95% confidence intervals across 20 replicate simulations. Solid grey lines show theoretical expectations. Also shown are scaling variances collapsed into a single category 'scl' and chromosome-level variance. Vertical dotted lines are placed to indicate the expected distance between recombination breakpoints that have accrued along a single chromosome since the hybridization pulse. In contrast to the figures shown in the main text, simulated loci are evenly spaced on a genetic map of the human autosomes, thus no interpolation is required and the estimates closely match the theoretical expectation.

bioRxiv preprint doi: https://doi.org/10.1101/2023.05.25.542345; this version posted May 26, 2023. The copyright holder for this preprint (which was not certified by peer review) is the author/funder, who has granted bioRxiv a license to display the preprint in perpetuity. It is made available under aCC-BY 4.0 International license.

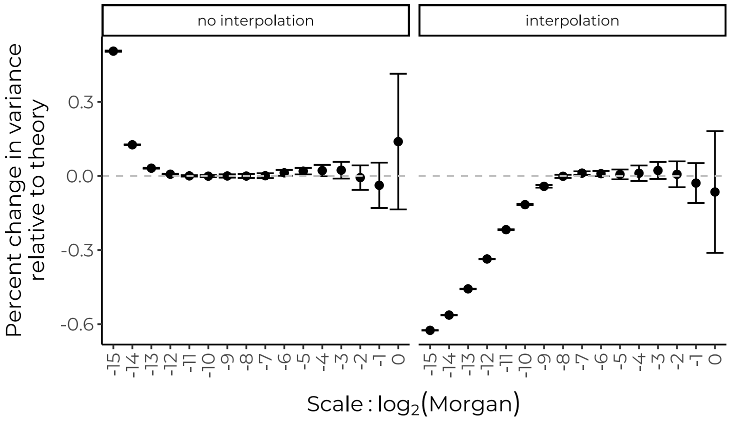

Figure S3: The effect of ancestry state interpolation on the power spectrum. Because informative markers are generally not evenly spaced, to perform the wavelet transform we must interpolate ancestry to evenly-spaced positions along the chromosome, either on a physical or genetic map. To see the scale of noise generated by interpolation, we compare simulated power spectra vs. theoretical expectations (Appendix B) for two cases after 1000 generations of mixture. **(Left)** Simulated loci are evenly spaced on the genetic map, thus no interpolation is required. In this case, deviations from the neutral expectation occur only at the finest scales, possibly reflecting numerical error. **(Right)** Simulated loci occur every 50kb on a physical map of the human autosomes, and ancestry is then interpolated to a grid on the genetic map to perform the wavelet transform. In this case, interpolation causes a downward bias over the left half of the power spectrum.

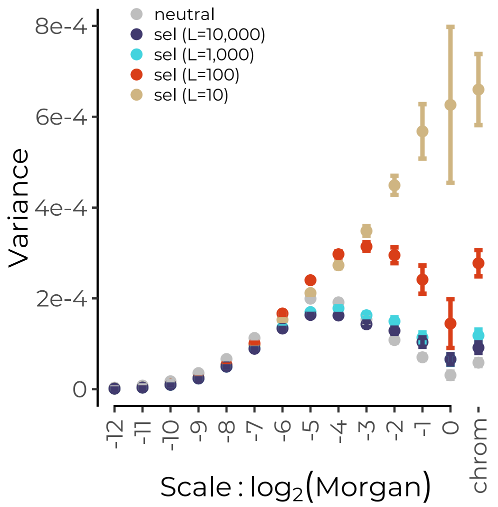

Figure S4: Selection against multiple alleles carried in one ancestry background distorts the wavelet variance decomposition of introgressed ancestry relative to the neutral expectation. We ran simulations of a 50/50 population mixture with selection acting against alleles from one ancestry at varying numbers of loci (10, 100, 1000, and 10000). In each case, the total strength of selection against F1 hybrids is held constant, where F1s have a 50% fitness reduction relative to the local source population. With fewer numbers of loci, there is stronger per locus selection concentrated in fewer regions, and selection at these loci generates increased broad-scale variation among regions harboring the deleterious loci and regions with mostly neutral variation.

bioRxiv preprint doi: https://doi.org/10.1101/2023.05.25.542345; this version posted May 26, 2023. The copyright holder for this preprint (which was not certified by peer review) is the author/funder, who has granted bioRxiv a license to display the preprint in perpetuity. It is made available under aCC-BY 4.0 International license.

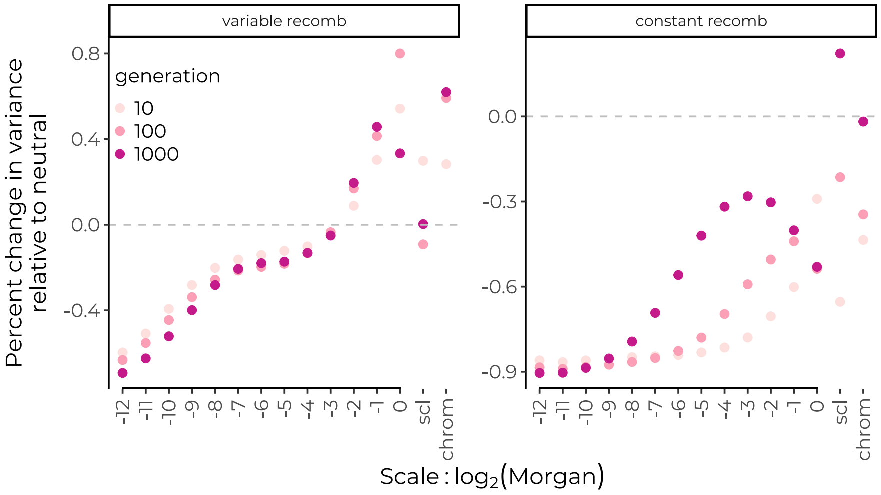

Figure S5: Genome-wide selection against alleles from one ancestry distorts the power spectrum. **(Left)** Selection on a heterogeneous recombination landscape modelled off the human autosomes generates greater variance at broad scales and lower variance at fine scales relative to the neutral expectation. Selection acts additively against alleles from one ancestry at 10,000 loci placed uniformly at random on the physical map, each selected allele has a selection coefficient of $s = 5e - 5$. The power spectrum of ancestry is compared to that from neutral simulations that use the same interpolation procedure. Thus, the distorion here is due to selection per se and not interpolation. The reduction in variance at fine scales after only 10 generations can be explained by the fact that selection brings the average introgressed ancestry proportion closer to zero. **(Right)** Here, simulated loci are placed evenly on a genetic map and results are compared to neutral simulations that do the same. Thus, although the overall variance is reduced, we see that selection distorts the power spectrum in a similar manner even in the absence of recombination rate heterogeneity.

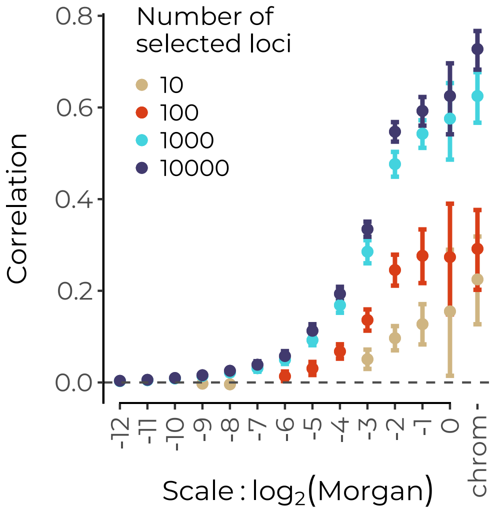

Figure S6: Selection against introgressed alleles generates correlations with recombination at varying scales according to the total number, and thus the spacing between selected loci. In simulations where the total strength of selection is held constant but distributed over varying numbers of loci, we find that in generation 1000, correlations with recombination are present at varying scales. With 10,000 loci under selection, fine scale recombination rate variation generates fine scale correlations between introgressed ancestry and recombination. With only 10 loci under selection, fine scale variation in recombination rate does not influence the rate of purging of deleterious introgressed alleles, so correlations at finer scales do not establish. Error bars represent 95% confidence intervals across 20 replicate simulations runs.

bioRxiv preprint doi: https://doi.org/10.1101/2023.05.25.542345; this version posted May 26, 2023. The copyright holder for this preprint (which was not certified by peer review) is the author/funder, who has granted bioRxiv a license to display the preprint in perpetuity. It is made available under aCC-BY 4.0 International license.

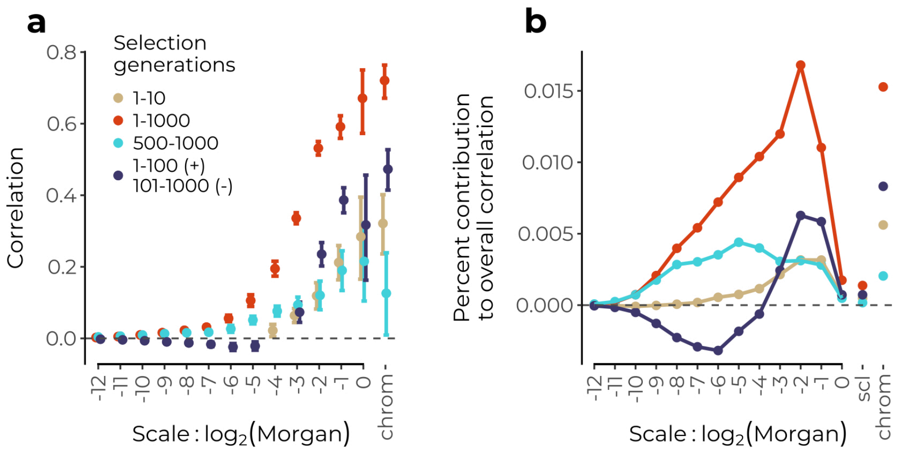

Figure S7: The wavelet decomposition of the correlation between recombination rate and introgressed ancestry can detect temporally-localized effects of selection in hybrids. **(a)** Selection acting only on the first 10 generations of recombinant hybrids generates significant positive wavelet correlations only at broad scales (brown) (viewed in generation 1000), whereas continuous selection over 1000 generations continues to generate correlations on finer scales (red). Selection that begins after generation 500 and of neutral mixture and continues until generation 1000 also generates fine-scale correlations, as well as broad-scale correlations. When selection acts continuously but reverses direction after 100 generations to favor the alternate ancestry, positive broad-scale correlations persist as negative correlations establish at finer scales. The comparison between these last two cases indicates that correlations at broad scales are dominated by the dynamics of selection in the early generations after mixture. Only significant correlations are shown, error bars represent 95% confidence intervals across 20 replicate simulations. **(b)** The correlations in **(a)** are weighted by the variance at each scale to give the contribution of each scale to the total correlation.

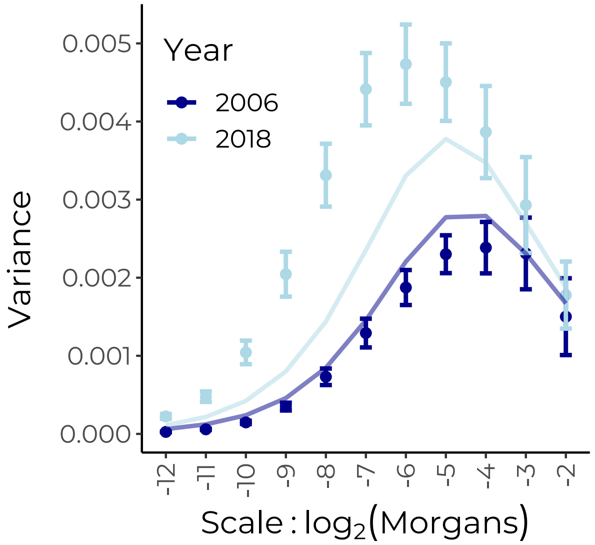

Figure S8: Power spectrum of proportion of malinche-like tracts in a hybrid swordtail population. Points show the empirical power spectra of proportion of malinche-like tracts in the two samples at the ends of the start and end of the time series. Error bars are 95% confidence intervals from a weighted block jacknife across chromosomes. Lines show theoretical expectations under neutrality, assuming 114 generations since initial admixture in the 2018 sample (previously estimate, Schumer lab), a generation time of 3 generations/year, and hybrid population size of 500 diploids, and an admixture proportion of 0.3.

bioRxiv preprint doi: https://doi.org/10.1101/2023.05.25.542345; this version posted May 26, 2023. The copyright holder for this preprint (which was not certified by peer review) is the author/funder, who has granted bioRxiv a license to display the preprint in perpetuity. It is made available under aCC-BY 4.0 International license.

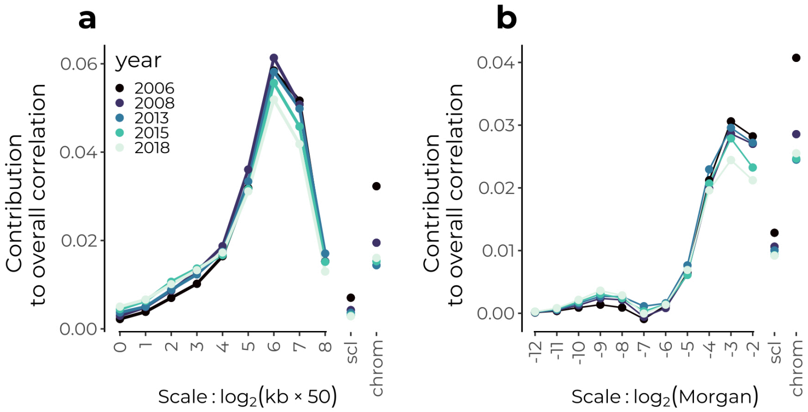

Figure S9: Contribution of each genomic scale to overall correlation between recombination and malinche-like ancestry. Results shown on the physical map **(a)** and genetic map **(b)**.

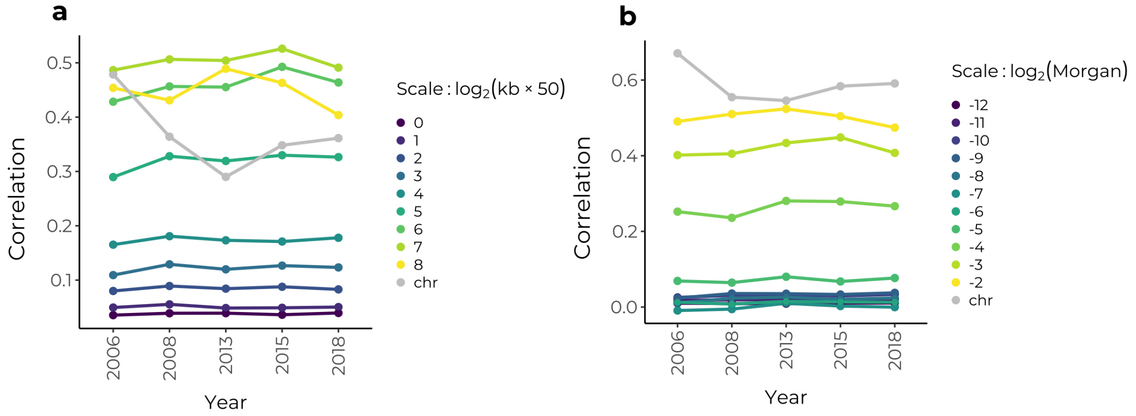

Figure S10: Correlations through time at varying spatial scales between recombination rate and minor parent ancestry (*X. malinche*). Results shown on the physical map **(a)** and genetic map **(b)**. Correlations between are not significantly different between any two timepoints at any scale.

bioRxiv preprint doi: https://doi.org/10.1101/2023.05.25.542345; this version posted May 26, 2023. The copyright holder for this preprint (which was not certified by peer review) is the author/funder, who has granted bioRxiv a license to display the preprint in perpetuity. It is made available under aCC-BY 4.0 International license.

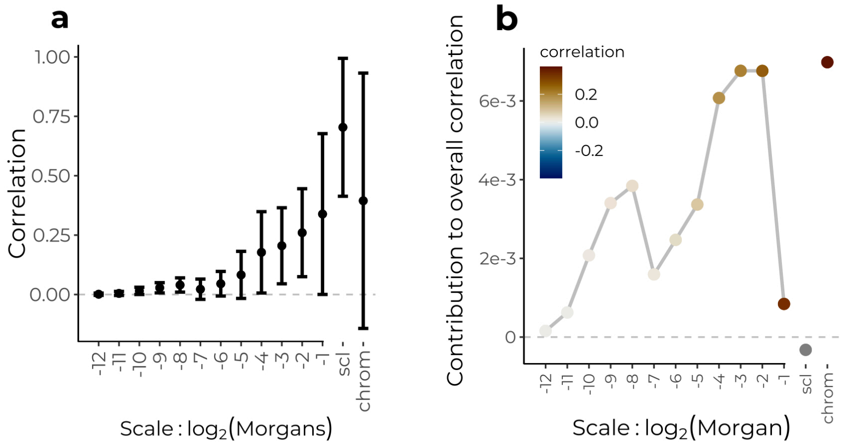

Figure S11: **(a)** Correlations across scales between recombination rate and anubis-like ancestry on the genetic map. **(b)** Contribution of each scale to the overall correlation (Eqn. 3).

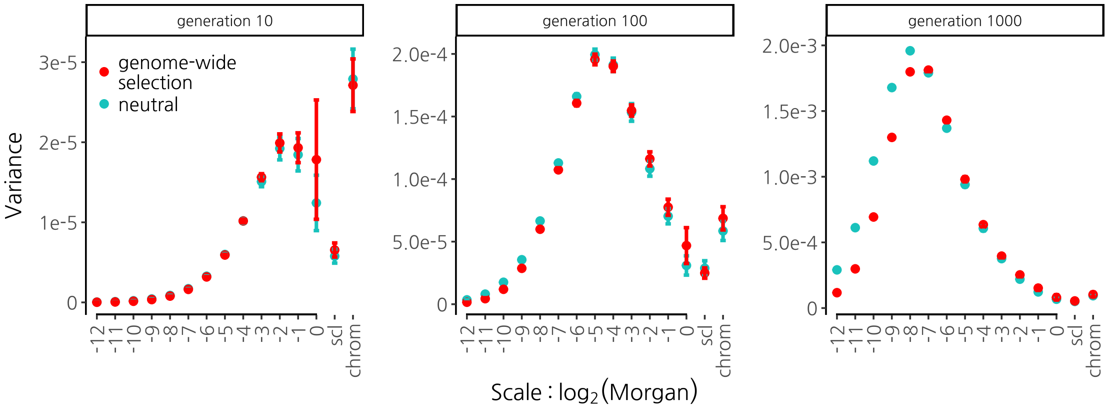

Figure S12: Power spectra of ancestry proportion for simulations with widespread weak selection (red) vs. neutral simulations (blue). Selection acts against alleles from one ancestry with additive effects within and across loci at 10,000 loci. Each allele has a selection coefficient of -2×10^{-5}, yielding a 20% fitness reduction in F1s. Error bars are 95% confidence intervals computed from 20 replicate simulations.

bioRxiv preprint doi: https://doi.org/10.1101/2023.05.25.542345; this version posted May 26, 2023. The copyright holder for this preprint (which was not certified by peer review) is the author/funder, who has granted bioRxiv a license to display the preprint in perpetuity. It is made available under aCC-BY 4.0 International license.

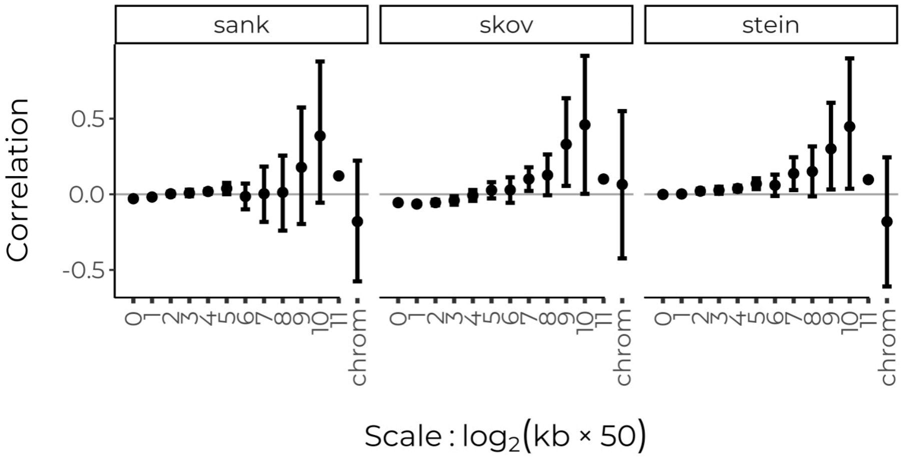

Figure S13: Correlations across scales between recombination rate and proportion of Neanderthal-like haplotypes on the human autosomes. **Left)** Data from Sankararaman *et al.* (2014) for the CEU sample of 1000 genomes. **Middle)** Data from Skov *et al.* (2020). **Right)** Data from Steinrücken *et al.* (2018) for the CEU population. Recombination rates are from Halldorsson *et al.* (2019) and are log10-transformed. Error bars show 95% confidence intervals from a weighted jackknife over chromosomes.

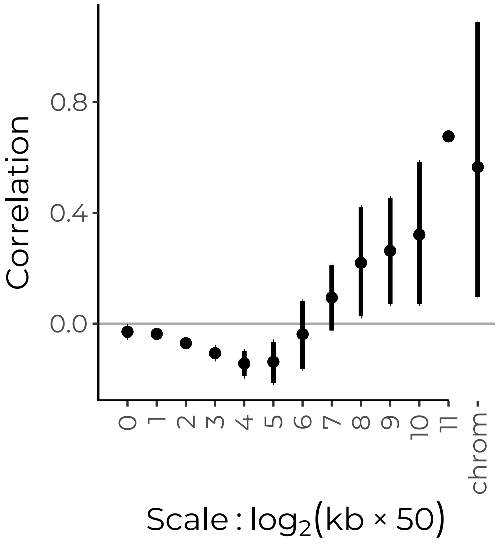

Figure S14: Correlations across scales between recombination rate and density of coding sequence base pairs on the physical map of the human autosomes.

bioRxiv preprint doi: https://doi.org/10.1101/2023.05.25.542345; this version posted May 26, 2023. The copyright holder for this preprint (which was not certified by peer review) is the author/funder, who has granted bioRxiv a license to display the preprint in perpetuity. It is made available under aCC-BY 4.0 International license.

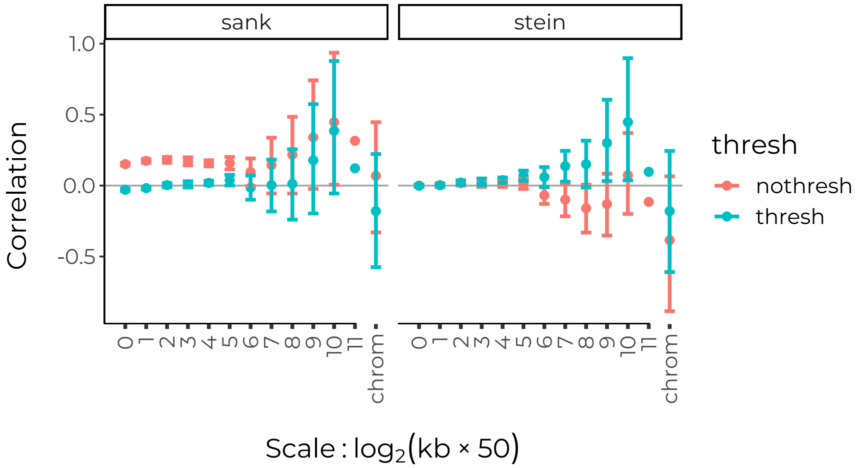

Figure S15: Effect of posterior threshold on correlations across scales between recombination rate and sample proportion of Neanderthal-like haplotypes estimated by Sankararaman *et al.* (2014) (left) and Steinrücken *et al.* (2018) (right). Following the methods described in these papers, we applied a thresholds of 0.9 and 0.42, respectively, to the posterior probabilities of a site matching a Neanderthal reference. We note that two studies that are commonly cited for evidence of selection against Neanderthal alleles utilize the non-thresholded posterior values from this study - Figure 2 in Sankararaman *et al.* (2014) and the work of Juric *et al.* (2016).
